# Defining molecular risk in ALK^+^ NSCLC

**DOI:** 10.18632/oncotarget.26886

**Published:** 2019-05-03

**Authors:** Petros Christopoulos, Jan Budczies, Martina Kirchner, Steffen Dietz, Holger Sültmann, Michael Thomas, Albrecht Stenzinger

**Affiliations:** ^1^ Department of Thoracic Oncology, Thoraxklinik and National Center for Tumor Diseases at Heidelberg University Hospital, Baden Württemberg, Heidelberg, Germany; ^2^ Institute of Pathology, Heidelberg University Hospital, Baden Württemberg, Heidelberg, Germany; ^3^ Division of Cancer Genome Research, German Cancer Research Center and National Center for Tumor Diseases, Baden Württemberg, Heidelberg, Germany; ^4^ Translational Lung Research Center Heidelberg, Member of the German Center for Lung Research, Baden Württemberg, Heidelberg, Germany

**Keywords:** ALK^+^ non-small cell lung cancer, *EML4-ALK* fusion variant, *TP53* mutation, treatment resistance, overall survival

## Abstract

Anaplastic lymphoma kinase (ALK)-positive non-small-cell lung cancers (NSCLC) have the best prognosis among metastatic pulmonary malignancies, with a median patient survival currently exceeding 5 years. While this is definitely a major therapeutic success for thoracic oncology, it may not be entirely attributable to rapid drug development and the strenuous clinical efforts. At the genetic level, ALK^+^ disease is also unique, distinguished by the lowest tumor mutational burden (mean below 3 mutations/Mbp), the lowest frequency of *TP53* mutations (20–25%) and very few other co-mutations compared to other NSCLC. The relative simplicity and stability of the genetic landscape not only contribute to the relatively favourable clinical course, but also make study of the effects from individual molecular features easier. *EML4-ALK* fusion variant 3 (E6;A20) and *TP53* mutations were recently identified as main molecular determinants of adverse outcome: they occur in about 30–40% and 20–25% of newly-diagnosed cases, respectively, have possibly synergistic effects and are independently associated with more aggressive disease, shorter progression-free survival under treatment with ALK inhibitors and worse overall survival. Secondary detection of *TP53* mutations at disease progression in previously negative patients defines another subset (about 20%) with similarly poor outcome, while detection of *ALK* resistance mutations guides next-line therapy. As our biological understanding deepens, additional molecular risk factors will be identified and refine our concepts further. The translation of clinical risk at the molecular level and the ability to predict early events are of key importance for individualized patient management and preclinical modeling in order to advance therapeutic options.

The question about molecular risk in anaplastic lymphoma kinase (ALK)-positive non-small cell lung cancer (NSCLC) is mainly posed by the recent therapeutic advances: prior to the availability of ALK tyrosine kinase inhibitors (TKI) and other targeted therapies, metastatic NSCLC was a rapidly lethal disease with a median overall patient survival (OS) below one and a half years [1]. In contrast, under sequential treatment with ALK TKI the median life expectancy of ALK^+^ lung cancer patients currently exceeds 5 years [2]. This impressive extension of patient life-span creates both the opportunity and the necessity to characterize early events, as their mechanistic understanding, prediction and tailored management will be crucial for further therapeutic progress.

Based on several retrospective analyses, it is well known that clinical parameters, e.g., advanced age [3], male sex [3], current smoking [2] and worse performance status [2], can predict worse survival of ALK^+^ NSCLC patients. These associations are plausible considering the naturally limited life expectancy of older individuals, the longer survival for women of any age [4], and the experience with other NSCLC, including EGFR^+^ lung adenocarcinoma, in which a positive smoking history and worse clinical condition at baseline are also associated with inferior outcome [5-7]. However, unfortunately, at the same time, predictive and prognostic implications of clinical variables are of limited utility, since clinical factors are neither a good source of mechanistic insights, nor informative for causal therapies that would improve the course of disease in individual patients.

Therefore, the translation of clinical profiles associated with higher risk into molecular features is an important, but challenging task in ALK^+^ NSCLC. Special obstacles include the rarity and genetic heterogeneity of the disease due to multiple *ALK* fusion variants [8], which are further potentiated by its complex management, including highly variable sequences of TKI and local ablative treatments [9], long patient survival of several years [2], limited availability of tissue from small biopsies, and variable ability of next-generation sequencing (NGS) assays to detect gene fusions in tissue or circulating tumor DNA (ctDNA) [10].

Recently, however, several studies combining state-of-the-art molecular profiling with detailed clinical annotation of large patient cohorts with long clinical follow-up identified two key molecular risk factors in ALK^+^ NSCLC: *EML4-ALK* fusion variant 3 (E6;A20) [11-16] and the presence of *TP53* mutations [17-19]. Among newly diagnosed patients, these genetic events occur independent from each other in about 30–40% and 20–25% of cases, respectively, have synergistic effects and are both associated with shorter progression-free survival (PFS) after treatment with first- and second-generation ALK inhibitors and with worse OS (Table 1) [11, 16, 18]. In addition, detection of *TP53* mutations in tissue or liquid rebiopsies at the time of disease progression in previously *TP53* negative patients, identifies another approximately 20% of cases with a poor outcome, comparable to that with primarily *TP53* mutated tumors (*TP53* status conversion in approximately 25% of cases ^x^ initially wildtype *TP53* result in approximately 75–80% of cases) [20].

**Table 1:** Baseline molecular risk in ALK^+^ NSCLC

	(% at diangosis)	Metastatic spread	PFS under TKI	OS
**V3**^**+**^	30-40%	↑^13,14,16,18^	↓^11,14,18 (12,16)^	↓^14,18 (16)^
***TP53***^**mut**^	20-25%	(depends on the oncogene)	↓^18,19 (31)^	↓^18,19 (17)^
***V3***^**+**^***TP53***^**mut**^	6-10%	↑↑^18^	↓↓^18^	↓↓^18^

Frequency of *EML4-ALK* V3 and *TP53* mutations in newly-diagnosed ALK^+^ NSCLC patients and their effect on main clinical characteristics of the disease, based on the collective insight from several studies [11-14,16-19,31].

Abbreviations: PFS: progression free survival, OS: overall survival; TKI: tyrosine kinase inhibitors.

Thus, the biology of ALK^+^ NSCLC displays some basic similarities with that of the other major oncogene-driven lung cancer subtype, namely EGFR^+^ NSCLC, in which the oncogene variant (e.g. exon 19 indels vs. other alterations [6]) and the presence of *TP53* mutations [21] influence benefit from TKI and patient survival, as well [22]. However, there are important differences. First, while in EGFR^+^ NSCLC the oncogene variants, such as exon 19 indels, L858R, “rare” point mutations and exon 20 insertions, cause a largely similar oncogenic drive [23-25], which nevertheless translates into a different clinical course only after institution of EGFR-directed therapies due to differential TKI sensitivity [26], the unfavourable *EML4-ALK* V3 variant in ALK^+^ NSCLC has a different biology *per se.* There is evidence that the shorter V3 oncoprotein is more stable [11, 27, 28], causes stronger ALK phosphorylation [11] and promotes cell motility and metastasis more efficiently [13, 16], resulting in a higher *a priori* clinical risk [29]. These data are supported by clinical observations: a higher incidence of metastatic disease [13] and as a higher number of metastatic sites in stage IV patients [14] at diagnosis, i.e. a more adverse course before and independent of the type of treatment (Figure 1A and Table 1) [16, 29]. Interestingly, in keeping with the finding of earlier and broader metastatic dissemination, the presence of V3 is also associated with a worse performance status of newly diagnosed ALK^+^ NSCLC patients (Figure 1B), which has already been recognized as a clinical risk factor in ALK^+^ disease [2], but is itself neither biologically insightful nor druggable. This observation further underlines the importance of defining disease risk at the molecular level in order to facilitate therapeutic advances. Of note, preclinical and limited clinical data suggest that besides *EML4-ALK* V3, other “short” *EML4-ALK* variants, such as V5 (E2;A20) [11], and non-*EML4-ALK* fusions [30] are also associated with worse outcome, while the longer *EML4-ALK* V2 (E20;A20) appears to be favourable [31]. The stronger kinase effects and weaker suppression of the V3 oncoprotein by first- and second-generation ALK inhibitors [11] may also facilitate earlier TKI escape through the development of *ALK* resistance mutations, which are another salient and clinically relevant characteristic of V3-driven disease [12]. Their occurrence depends not only on the type of *ALK* fusion, but also on TKI sequencing and is important for the choice of next-line therapy [9, 32, 33]. A recently published analysis in a subset (for example 37% or 112/303 cases with detection of V1, V2, V3 in tissue) of patients from the ALEX trial suggested a superior outcome with alectinib compared to crizotinib for patients with all three main *EML4-ALK* fusion variants V1, V2, V3, but also a trend that the benefit from alectinib, i.e. the response rate (*p* = 0.10) and the PFS (*p* = 0.11), might be lower in case of non-V1 *EML4-ALK* variants, as detected in tissue samples [34]. Since these data are still immature (based on the data cut-off of December 2017), and the patient subgroups are small (*n* = 8-25, i.e. smaller than in previous retrospective analyses), these results have to be interpreted with some caution.

**Figure 1:**
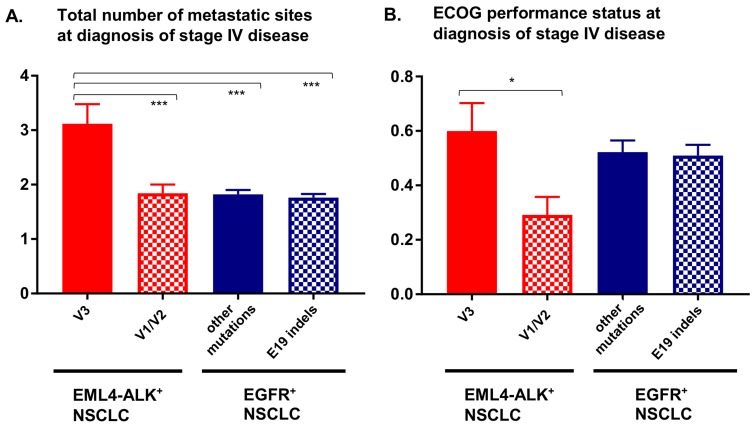
Number of metastatic sites and performance status in newly diagnosed stage IV EML4-ALK^+^ and EGFR^+^ NSCLC patients **A.** The total number of metastatic sites (intrathoracic, brain, liver, bone, adrenal, other) for newly diagnosed stage IV EML4-ALK^+^ (*n* = 34 V3 cases, *n* = 44 V1/V2 cases) and stage IV EGFR^+^ (*n* = 221 *EGFR* exon 19 [E19] indel cases, *n* = 197 cases with other *EGFR* mutations) NSCLC patients typed at our institution with available data [18,22]. Statistical comparison was performed with ANOVA (*p* < 0.001) followed by the Dunnett’s post-hoc test. Columns and error bars show mean values and their standard errors: 3.12 and 0.36 for *EML4-ALK* V3, 1.84 and 0.16 for *EML4-ALK* V1/V2, 1.76 and 0.07 for *EGFR* exon 19 indels, 0.82 and 0.08 for other *EGFR* alterations. Statistically significant results are shown in the graph; ***: *p* < 0.001. **B.** The Eastern Cooperative Oncology Group (ECOG) performance status for newly diagnosed stage IV EML4-ALK^+^ (*n* = 35 V3 cases, *n* = 48 V1/V2 cases) and EGFR^+^ (*n* = 210 *EGFR* exon 19 [E19] indel cases, *n* = 178 with other *EGFR* mutations) NSCLC patients from our institution with available data [18,22]. Statistical comparison was performed with ANOVA (*p* < 0.05) followed by the Dunnett’s post-hoc test. Columns and error bars show mean values and their standard errors: 0.60 and 0.10 for *EML4-ALK* V3, 0.29 and 0.07 for *EML4-ALK* V1/V2, 0.52 and 0.04 for *EGFR* exon 19 indels, 0.51 and 0.04 for other *EGFR* alterations); *:*p* = 0.037.

A second major difference that distinguishes ALK-driven lung cancers from their EGFR^+^ counterparts is an apparently even lower genetic complexity based on a very low tumor mutational burden (TMB, mean 2.0 *vs*. 5.0 mutations/Mbp in the MSKCC cohort [35-37], *p* < 0.001, Figure 2A), a lower frequency of *TP53* mutations (25% *vs.* 42%, *p* < 0.01, Figure 2B, 2C) [18, 19, 35], and few other co-mutations (Figure 2D) [19, 22]. Of note, both the very low TMB (mean < 3, Figure 2A) and the very low *TP53* mutation rate (about 20–25%, Figure 2B) are unique features of ALK^+^ tumors distinguishing them from all other NSCLC [18, 38]. Since TMB of oncogene-driven lung cancer is higher in the presence of *TP53* mutations (Figure 2C), the two parameters appear to be linked, possibly through DNA damage facilitated by *TP53* loss due to genetic instability [39]. Such an accumulation of genetic abnormalities is crucial for the development of TKI failure [40-42], which is associated not only with the presence of *TP53* mutations [21], but also with a higher TMB in EGFR^+^ NSCLC [43]. For example, specific co-occuring genetic alterations acquired with disease progression (e.g. in *CTNNB1* and *PIK3CA*), were shown to cooperatively promote tumor metastasis, while other evolutionary paths impair the apoptotic response and cause resistance to EGFR inhibitors [44, 45]. According to very recent data on *HER2*-amplified gastroesophageal adenocarcinoma, increased genomic complexity can reduce efficacy of targeted therapies even when more than one target is hit at the same time [46, 47]. Future studies are warranted to analyze whether this observation can be conceptualized: is the degree of intratumoral heterogeneity a predictor of response to targeted drugs across cancer types independent of the druggable target itself [48]? Conversely, the lower baseline TMB and *TP53* mutation rate of ALK^+^ compared to EGFR^+^ NSCLC (Figure 2A, 2B) suggest a more “benign” biology. Consistent with this notion, ALK^+^ NSCLC patients treated with even just one TKI have a longer OS than TKI-treated EGFR^+^ NSCLC patients (Figure 3, Table 2), and survival has generally been longer in trials of ALK inhibitors compared to trials of EGFR inhibitors (for example median OS was > 45 months in the Profile 1014 trial of the first-generation ALK inhibitor crizotinib [49] vs. 28 months in the LUX-Lung-3 study of the second-generation EGFR inhibitor afatinib [50]). This lower genetic complexity of ALK-driven NSCLC compared to other lung tumors may explain why therapeutic progress has been much faster and survival gains much larger for ALK^+^ compared to other lung cancer patients. Interestingly, the worse outcome of smoker ALK^+^ and EGFR^+^ NSCLC patients [2, 6] appears to correlate with a higher TMB [51], which illustrates again how “traditional” clinical risk factors can be redefined at the molecular level in order to promote deeper understanding of basic pathogenetic processes.

**Figure 2:**
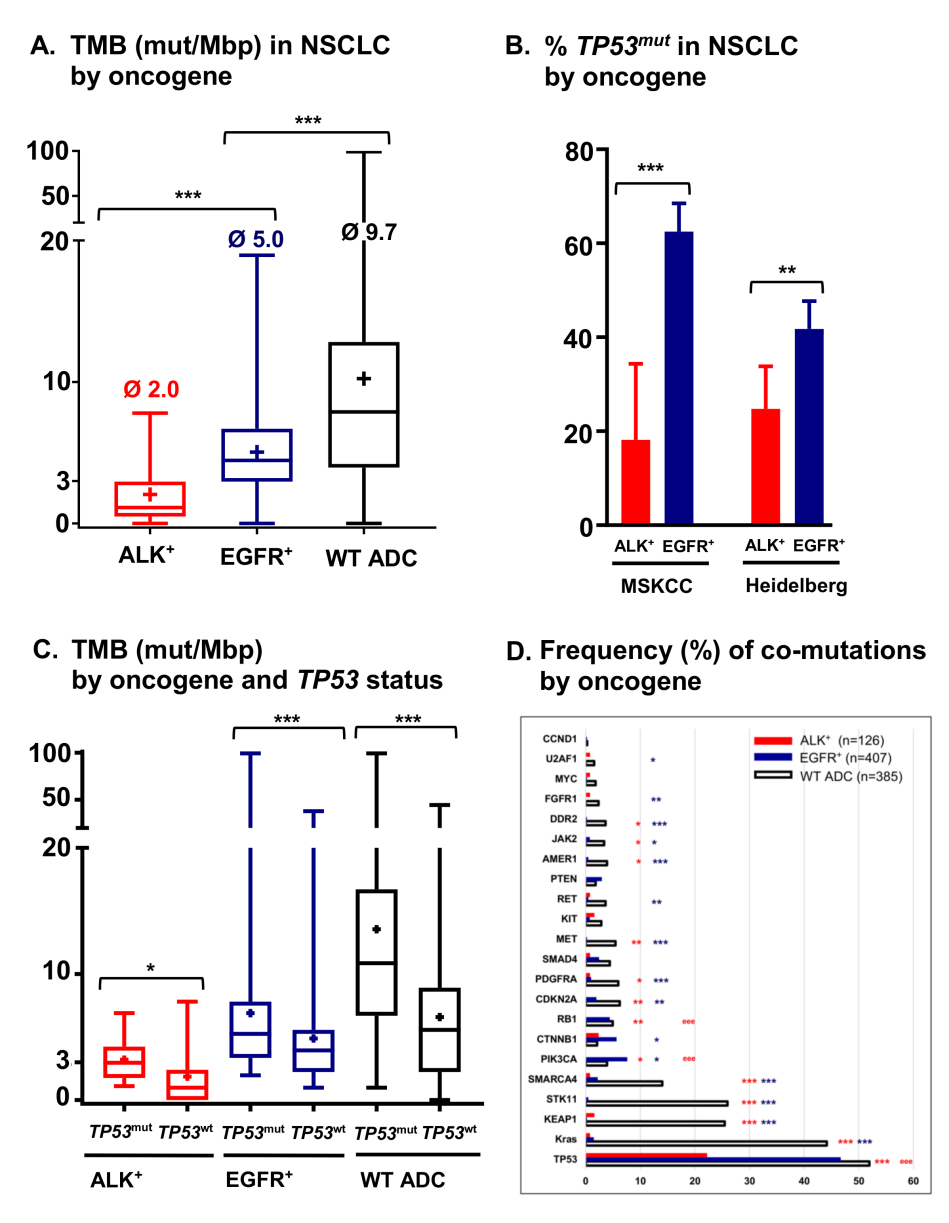
Tumor mutational burden, frequency of TP53 mutations and frequency of co-mutations in metastatic ALK^+^ and EGFR^+^ NSCLC **A.** Tumor mutational burden (TMB) of metastatic ALK^+^ (*n* = 33, mean 2.0 mutations[mut]/Mbp), EGFR^+^ (*n* = 232, mean 5.0 mut/Mbp) and wildtype (WT, i.e. ALK/EGFR/RET/ROS-negative, *n* = 557, mean 9.7 mut/Mbp) cases from the publicly available MSKCC lung adenocarcinoma (ADC) cohort (http://www.cbioportal.org) as estimated by targeted sequencing with the IMPACT341 and IMPACT411 panels [35-37]. For cases with multiple sampling time-points, only the earliest one in the disease course was analyzed, and among multiple samples at the earliest time-point, that with the highest number of mutations was chosen. Boxplots show medians, means (“+”) and range; ***p < 0.001 with the Kruskal-Wallis test followed by the Dunn’s post-hoc test. **B.** Frequency of *TP53* mutations in metastatic ALK^+^ (18%, *n* = 33) and EGFR^+^ (63%, *n* = 232) NSCLC cases of the MSKCC cohort [35-37], as well as in untreated metastatic ALK^+^ (25%, *n* = 105) and EGFR^+^ (42%, *n* = 273) tumors sequenced for exons 4-10 of *TP53* at our institution [18, 22]. Columns and error bars show percentages and 95% confidence intervals, respectively; ***p < 0.001, and **p = 0.0022 with a chi-square test. **C.** Tumor mutational burden (TMB) according to *TP53* status for ALK^+^ (mean 3.2 mut/Mbp for *TP53* mutated, *n* = 6, *vs.* 1.8 mut/Mbp for *TP53* wildtype cases, *n* = 27, *p* = 0.039 with a Mann-Whitney test), EGFR^+^ (mean 5.6 mut/Mbp, *n* = 145, *vs.* 4.0 mut/Mbp, *n* = 87, *p* < 0.001) and WT cases (mean 13.6 mut/Mbp, *n* = 291, *vs.* 6.6 mut/Mbp, *n* = 266, *p* < 0.001) from the publicly available MSKCC lung adenocarcinoma cohort (http://www.cbioportal.org) [35-37]. In a bivariable linear regression analysis among ALK^+^ and EGFR^+^ patients, type of oncogene (*EGFR vs. ALK*, beta = 0.248, *p* < 0.001) and *TP53* status (mutated *vs*. wild-type, beta = 0.256, *p* < 0.001) were similarly strong determinants of TMB. Boxplots show medians, means (“+”) and range. **D.** Frequency of co-mutations in untreated ALK^+^, EGFR^+^ and WT NSCLC patients. Analyzed were untreated ALK^+^ (*n* = 105) and EGFR^+^ (*n* = 273) patients sequenced with PCR-based DNA NGS using our custom panel of 38 genes as previously described [22], as well as the untreated ALK^+^ (*n* = 21), EGFR^+^ (*n* = 134) and WT (*n* = 385) patients of the MSKCC lung adenocarcinoma cohort sequenced with the MSK-IMPACT341 and MSK-IMPACT411 panels (http://www.cbioportal.org) [35-37]. Visualized are all common genes among the three panels with at least one detectable mutation. Statistical comparisons for ALK^+^ *vs.* WT, and EGFR^+^ *vs*. WT were performed with a chi-square test, and results with *p* < 0.05 and Benjamini-Hochberg *q* < 0.05 are shown in the graph in red and dark blue color, respectively: *:*p* < 0.05, **:*p* < 0.01, ***:*p* < 0.001. Statistical comparison for ALK^+^ vs. EGFR^+^ was performed similarly, and significant results are shown in red color: ^e^:*p* < 0.05, ^ee^:*p* < 0.01, ^eee^:*p* < 0.001.

**Figure 3:**
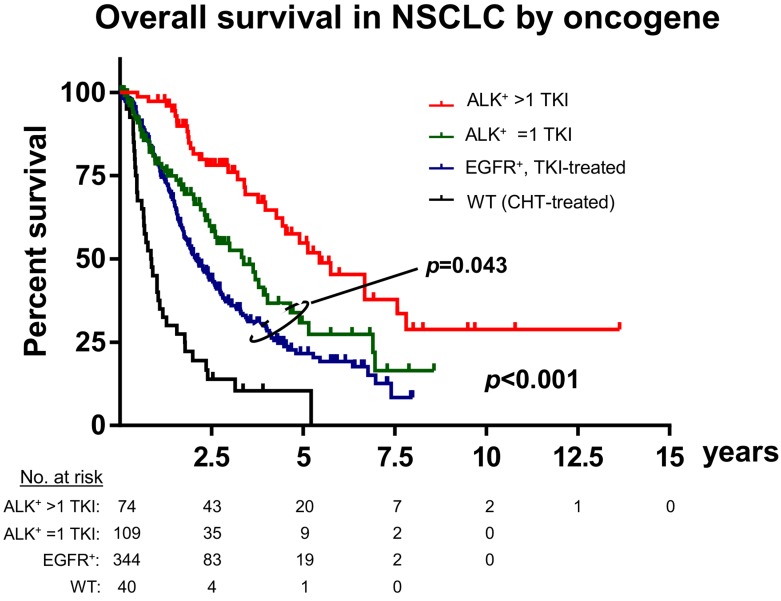
Differential outcome of ALK^+^, EGFR^+^ and chemotherapy-treated wildtype NSCLC Retrospective analysis of tyrosine kinase inhibitor (TKI)-treated ALK^+^ (*n* = 74 with >1 TKI, *n* = 109 with just 1 TKI) and EGFR^+^ (*n* = 344) NSCLC patients, along with a random sample of *n* = 40 EGFR/ALK-wildtype NSCLC patients that received chemotherapy in the Thoraxklinik at Heidelberg University Hospital [22]. Basic clinical and treatment characteristics of ALK^+^ and EGFR^+^ patients are shown in Table 2. Median overall survival was 65 months for ALK^+^ patients that received >1 TKI, 40 months for ALK^+^ patients that received just 1 TKI, 25 months for TKI-treated EGFR^+^ patients and 10 months for wildtype patients; *p* < 0.001 across groups and *p* = 0.043 for the comparison between the EGFR^+^ and ALK/1-TKI subgroups with a log-rank test; WT: wildtype; CHT: chemotherapy.

**Table 2:** Characteristics of the ALK^+^ and EGFR^+^ NSCLC patients included in Figure 3

ALK^+^ patients	ALK^+^ NSCLC			EGFR^+^ NSCLC TKI-treated, (*n* = 344)^3^	
	>1 TKI, (*n* = 74)		1 TKI, (*n* = 109)		
Age (median; IQR)	51; 14		59; 11	65; 12	
Never/light-smokers (<10 py), n (%) ^1^	44/50 (88%)		53/78 (68%)	154/341 (45%)	
ECOG PS (median; IQR) ^1^	0; 1		0; 1	1;1	
TKI treatment, n (%)	crizotinib	72 (97%)	92 (85%)	erlotinib	183 (53%)
	ceritinib	50 (68%)	7 (6%)	gefitinib	79 (23%)
	alectinib	46 (62%)	8 (7%)	afatinib	117 (34%)
	brigatinib	14 (19%)	2 (2%)	osimertinib	61 (18%)
	lorlatinib	6 (8%)	-		
Summary of the complete treatment					
no. of TKI treatment lines (mean; SD)	2.6; 0.8		1.0; 0.0	1.3; 0.6	
no. of treatment lines for St. IV (mean; SD)	4.2; 1.6		2.3; 1.6	2.2; 1.3	
patients with additional radiotherapy (%)	43 (58%)		48 (44%)	176 (51%)	
patients with additional surgical treatment ^2^	9 (12%)		10 (9%)	25 (7%)	

^1^ Data not available for all patients.

^2^ Excluding video-assisted thoracoscopy for pleural effusion.

^3^ Most patients were treated before availability of osimertinib, and some received more than one EGFR inhibitors, due to poor tolerability of the first compound or in different treatment lines alternating with chemotherapy.

Abbreviations: IQR: interquartile range; py: pack-years; PS: performance status; TKI: tyrosine kinase inhibitors; SD: standard deviation; no.: number.

The scarcity of additional genetic alterations is presumably an important reason why *TP53* mutations have a major effect on the clinical phenotype of ALK^+^ NSCLC: they are associated with increased disease aggressiveness and metastatic dissemination synergistically with *EML4-ALK* V3, and they are linked with shorter PFS under TKI and shorter OS independently from *EML4-ALK* V3, so that double positive V3^+^*TP53*^+^ patients have a very high risk of death with a median OS of around 2 years in our series (Table 1) [18]. In contrast, a predictive and prognostic impact of *TP53* mutations has been hard to discern in case of EGFR/ALK-negative NSCLC [52], in which the much higher number of genetic alterations at baseline [53] presumably obscures the effect of *TP53* status and dilutes the consequences of genetic instability [39]. A analogous difference becomes apparent if solid cancers [54, 55] are collectively considered against hematologic malignancies, for example acute myeloid leukemia, multiple myeloma, chronic lymphocytic leukemia and mantle-cell lymphoma: in the latter TMB is generally lower (median <3 mutations/Mbp) [56] and *TP53* abnormalities are less frequent (generally <10-15%), but more important for clinical course and crucial for patient management [57-60]. In the model disease of precision medicine, chronic myeloid leukemia, *TP53* abnormalities and other cytogenetic aberrations or co-mutations are also associated with clonal evolution, TKI failure and poor outcome [61-63]. Transgenic mouse models of oncogene-, for example *Kras-* or *Egfr*-driven NSCLC, demonstrate this principle nicely through a paucity of concomitant genetic alterations [64], but a dramatic phenotypic change upon *TP53* loss with metastases and earlier death [65]. Presumably along the same lines, *TP53* alterations impair outcome of ALK^+^ NSCLC patients more in case of TKI treatment compared to chemotherapy [18], which is itself genotoxic [66].

Thus, although scarce, concomitant genetic alterations appear to be another important determinant of tumor biology and patient outcome in ALK-driven NSCLC, beside the oncogenic driver, i.e. the *ALK* fusion itself. For example, *KRAS* amplifications promote resistance to ALK inhibitors by activating RAS-MAPK signaling [67], which is amenable to SHP2 inhibition [68]. A similar picture emerges for EGFR^+^ NSCLC, in which however the spectrum of oncogene alterations and co-mutations is much broader [43, 69]. The independent and possibly synergistic effects of both the *ALK* fusion variant and *TP53* mutations (as well as other, yet to be identified molecular features) on the clinical course of ALK^+^ NSCLC patients [18] mean that considerable biological and clinical variability is to be expected, if a study would take only one of these molecular factors into account. For effective guidance of patient management that is based on the molecular properties of the tumor, broad profiling approaches will be required, which could for example utilize combined targeted RNA and DNA NGS [22].

From a clinical standpoint, there is currently little that can be done for higher-risk, i.e., V3^+^, *TP53*^mut^ and particularly V3^+^*TP53*^mut^ ALK^+^ NSCLC patients. When discussing life-expectancy, some reservation is warranted, especially for “double-positive” V3^+^*TP53*^mut^ cases, for which the 5-year landmark does not apply [18]. At the time of disease progression, a more aggressive strategy regarding local ablative therapies should be considered, otherwise some of these patients can rush through all available ALK TKI lines, for example with a change of therapy every 4-6 months, and end up with palliative chemotherapy within the first 2 years [18]. More frequent radiologic surveillance, additional ctDNA monitoring [20], upfront administration of more potent ALK inhibitors and combination with experimental compounds, such as *TP53*-directed drugs [70] may also be beneficial, but their clinical utility needs to be tested in prospective trials. Moreover, *in vitro* and animal studies of the tumor-promoting effects from different *ALK* fusion variants and *TP53* mutations in ALK^+^ disease will be instrumental for deeper mechanistic insights towards uncovering of therapeutic susceptibilities and new drug development.

In summary, ALK^+^ NSCLC is currently spearheading the advent of “precision medicine” in thoracic oncology [11-14, 16, 18, 19, 27, 28, 30]. Distinguished by the lowest genetic complexity and the longest patient survival among NSCLC, ALK^+^ disease is serving as a model for the illustration of basic biological principles and for the development of novel therapeutic strategies, which will probably prove useful in other lung cancer subtypes and tumor entities as well. As our understanding of its pathogenesis deepens, additional molecular features critical for patient outcome will be identified and used to further refine our concepts. Elaboration of a baseline molecular risk stratification—complemented by profiling and targeting of treatment resistance—is a crucial step towards tailored, more effective patient management and better preclinical modeling that will foster therapeutic progress.
